# Spatial heterogeneity of hotspot mutations and clonal relatedness in ovarian endometrioid carcinoma

**DOI:** 10.1002/1878-0261.70347

**Published:** 2026-10-08

**Authors:** Takahito Ashihara, Kosuke Murakami, Shiro Takamatsu, Akiko Kanto, Seiichi Mori, Tomoyuki Otani, Kazuko Sakai, Yasushi Kotani, Kazuto Nishio, Kaoru Abiko, Noriomi Matsumura

**Affiliations:** ^1^ Department of Obstetrics and Gynecology Kindai University Faculty of Medicine Osaka Japan; ^2^ Department of Obstetrics and Gynecology Kyoto Katsura Hospital Kyoto Japan; ^3^ Project for Development of Innovative Research on Cancer Therapeutics, Cancer Precision Medicine Center Japanese Foundation for Cancer Research Tokyo Japan; ^4^ Department of Pathology Yachiyo Hospital Aichi Japan; ^5^ Department of Genome Biology Kindai University Faculty of Medicine Osaka Japan; ^6^ Department of Obstetrics and Gynecology Kanazawa University Graduate School of Medical Sciences Ishikawa Japan

**Keywords:** clonal relatedness, droplet digital PCR, laser capture microdissection, ovarian endometrioid carcinoma, spatial heterogeneity, synchronous endometrial and ovarian carcinomas

## Abstract

Ovarian endometrioid carcinoma (OEC) is a major histotype of endometriosis‐associated ovarian carcinoma, yet the spatial distribution of cancer‐associated mutations and the clonal relatedness of synchronous endometrial and ovarian carcinomas (SEOC) remain incompletely understood. In this study, we performed spatially controlled molecular profiling of OEC using laser capture microdissection, droplet digital PCR, targeted amplicon sequencing, and exploratory whole‐exome sequencing. We analyzed multiple tumor regions and selected stromal compartments from OEC and SEOC cases to assess the intratumor distribution of hotspot mutations in *CTNNB1*, *PIK3CA*, and *KRAS* and to evaluate molecular relatedness between ovarian and endometrial lesions in SEOC. Cancer‐associated mutation signals showed spatially heterogeneous distributions in a subset of histologically uniform OEC. In SEOC, shared alterations supported clonal relatedness in selected cases. Low‐level hotspot mutation signals were also detected in microdissected stromal compartments immediately adjacent to tumor glands, although their cellular origin remains to be determined. These findings suggest that single‐region molecular testing may underestimate spatial molecular heterogeneity in OEC and provide a framework for understanding the clonal relatedness of endometriosis‐associated gynecologic carcinomas.

## Graphical abstract

Spatially resolved analysis revealed heterogeneous distributions of selected cancer‐associated mutations within ovarian endometrioid carcinomas. Shared mutations between paired endometrial and ovarian tumors supported clonal relatedness. These findings highlight the value of sampling multiple tumor regions when interpreting molecular profiles. Abbreviations: OEC, ovarian endometrioid carcinoma; SEOC, synchronous endometrial and ovarian carcinomas; LCM, laser capture microdissection.

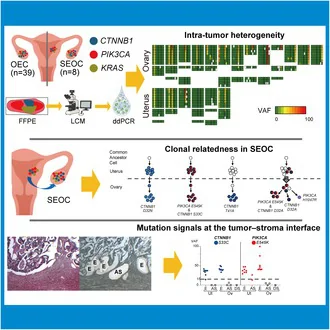

**Abbreviations**
LCM: laser capture microdissection
LOH: loss of heterozygosity
MMR: mismatch repair
OCCC: ovarian clear cell carcinoma
OEC: ovarian endometrioid carcinoma
SEOC: synchronous endometrial and ovarian carcinomas
UEC: uterine endometrioid carcinoma
VAF: variant allele frequency

## Introduction

The incidence of ovarian cancer is globally increasing [1]. Among epithelial ovarian cancers, ovarian endometrioid carcinoma (OEC) is a type of ‘endometriosis‐associated ovarian cancer’ that occurs more frequently in women with a history of or concurrent endometriosis, along with ovarian clear cell carcinoma (OCCC) [2, 3, 4, 5]. Endometriosis primarily results from retrograde menstruation of endometrial tissue, and endometriosis‐associated ovarian cancers arise from endometrial tissue [3, 5, 6, 7, 8, 9, 10]. Consequently, OEC often shares cancer‐associated gene mutations with uterine endometrioid carcinoma (UEC), and these tumors can exhibit overlapping molecular pathologies [11, 12, 13, 14]. In addition, OEC is occasionally accompanied by synchronous endometrial cancer, most commonly UEC; this condition is referred to as synchronous endometrial and ovarian carcinomas (SEOC) [15, 16]. Historically, endometrial and ovarian tumors in SEOC were considered separate primary neoplasms. However, recent genetic analyses have shown that many SEOCs are clonally related [17, 18, 19] and may represent metastatic dissemination between the endometrium and ovary rather than independent primary tumors [7, 9, 10]. Nevertheless, the distribution of genetic mutations within the tumors and at the tumor–stroma interface in these two types of tumors—which are similar and sometimes occur synchronously—has not been fully elucidated.

Recent advances in genomic analysis enable the analysis of genetic mutations in micro‐regions within tumor tissues, leading to the accumulation of new insights into tumor clonal evolution and the boundaries between tumor and surrounding tissues. Among these analytical methods, the development of spatially resolved sequencing has been remarkable. However, spatially resolved DNA sequencing still faces limitations, such as the difficulty of using formalin‐fixed paraffin‐embedded (FFPE) samples and restrictions on single nucleotide variant (SNV) identification [20, 21]. To overcome these issues, a combination of laser capture microdissection (LCM) with whole‐genome/exome sequencing or targeted sequencing has been employed [22, 23]. Moreover, oncogenic driver mutations in cancer are often concentrated in specific hotspots [24, 25, 26, 27, 28], and if the target genes are identified, droplet digital PCR (ddPCR) can be used to detect low‐frequency mutations with high sensitivity, in a short time, and at low cost, while preserving spatial information, even with very small DNA samples [29, 30, 31]. Previously, our group combined LCM and ddPCR to demonstrate the spatial heterogeneity of *PIK3CA* mutations within OCCC tumors [32]. In contrast, no previous studies have demonstrated the spatial distribution of genetic mutations within OEC or SEOC tumors. Additionally, in OEC, hotspot mutations in *CTNNB1* and *KRAS* occur at higher frequencies than in OCCC [13, 14], making ddPCR analysis more feasible.

In this study, we performed spatially controlled, multiregion molecular profiling of ovarian endometrioid carcinoma, including SEOC, using LCM, ddPCR, targeted amplicon sequencing, and exploratory whole‐exome sequencing. We aimed to clarify the spatial distribution of cancer‐associated mutations within histologically defined tumor regions, assess clonal relatedness between synchronous endometrial and ovarian tumors, and explore mutation signals at the tumor–stroma interface. By integrating histopathological compartmentalization with sensitive mutation detection, this study provides insight into the spatial distribution of cancer‐associated mutations in endometriosis‐associated gynecologic carcinomas.

## Materials and methods

### Patients and samples

Between January 2008 and December 2020, 39 patients who were surgically treated at Kindai University Hospital (Osaka, Japan) and were diagnosed with OEC (including eight cases of SEOC) were included. In addition, three patients with UEC and concomitant endometrial hyperplasia, diagnosed at the Cancer Institute Hospital (Tokyo, Japan), were included (Fig. S1). Histological diagnoses were reviewed by gynecologic pathologists according to the World Health Organization classification. Clinicopathological information, including age, the International Federation of Gynecology and Obstetrics (FIGO) stage, tumor grade, and immunohistochemical findings, was retrieved from medical records and pathology reports. Cases with sufficient formalin‐fixed paraffin‐embedded tissue for LCM and molecular analysis were included. SEOC cases were diagnosed based on clinicopathological criteria, including tumor location, histological similarity, depth of myometrial invasion, presence or absence of endometriosis, ovarian surface involvement, lymphovascular invasion, and extrauterine disease. Molecular findings were used to assess clonal relatedness but were not used as the sole basis for the initial clinicopathological diagnosis.

This study was approved by the Institutional Review Board of Kindai University Faculty of Medicine (approval number: R03‐180) and was conducted in accordance with the Declaration of Helsinki. The requirement for written informed consent was waived by the Institutional Review Board because of the retrospective nature of the study. Information about the study was posted on the institutional website (https://www.kindai.ac.jp/medicine/), and patients were given the opportunity to opt out.

### Macrodissection and DNA extraction

For each OEC sample, the tumor epithelial region and stroma were identified using hematoxylin and eosin (HE) staining. Following this, macrodissection was performed on 5‐μm‐thick FFPE sections to isolate the tumor epithelial region. The samples were deparaffinized, and DNA was extracted using the QIAamp DNA Micro Kit (Qiagen, Hilden, Germany) according to the manufacturer's protocol. Additionally, in three cases of endometrial cancer with concomitant endometrial hyperplasia, samples were collected from fresh‐frozen specimens and divided into endometrial cancer, stroma adjacent to the tumor epithelium, atypical endometrial hyperplasia, and stroma adjacent to atypical endometrial hyperplasia. DNA was extracted using the same protocol as described above.

### Laser capture microdissection and DNA extraction

Laser capture microdissection was performed on formalin‐fixed paraffin‐embedded tissue sections using a Leica LMD system. Tumor epithelial regions and selected stromal compartments were identified on HE‐stained sections. For the tumor epithelial and stromal regions of OEC and SEOC, regions containing approximately 20–30 cells were collected using LCM, as previously reported [32]. Briefly, FFPE was sliced into 10‐μm‐thick sections, mounted on foil‐coated slides (Leica, Wetzlar, Germany), stained with toluidine blue, and dissected using a Leica LMD7000 (Leica) (Fig. S2). Multiple spatially distinct tumor regions were sampled from each case when available. Histologically uniform tumor areas were defined as regions showing similar endometrioid glandular architecture and cytological features on serial histological sections. Adjacent stromal compartments were defined as stromal areas immediately adjacent to the tumor glands (< 200 μm) but morphologically distinct from them. The regions for LCM were defined on serial HE‐stained sections. After microdissection, the postdissection slide was compared with the corresponding serial HE section, and a gynecologic pathologist (T.O.) confirmed that the adjacent and distant stromal compartments excluded overt tumor epithelium. Distant stromal compartments, when available, were sampled from areas separated from invasive tumor glands (≥ 1000 μm) and used as comparative nonepithelial compartments. For each case, up to eight samples were collected from the tumor epithelial region, up to four samples from the stroma region adjacent to the tumor epithelial region, and up to four samples from the stroma region distant from the tumor epithelial region. DNA was extracted using the same protocol as described above.

### Targeted amplicon sequencing for macrodissection samples

For DNA sequencing, 10 nanograms of DNA were subjected to multiplex PCR amplification with the use of an Ion AmpliSeq Library Kit 2.0 and Ion AmpliSeq Cancer Hotspot Panel v2 (Life Technologies, Carlsbad, CA, USA). After multiplex PCR, Ion Xpress Barcode Adapters (Life Technologies) were ligated to the PCR products, which were then purified using Agencourt AMPure XP beads (Beckman Coulter, Brea, CA, USA). The purified libraries were pooled and then sequenced using an Ion S5 XL system and an Ion 550 Chip (all from Thermo Fisher Scientific, Waltham, MA, USA). DNA sequencing data were accessed through the Torrent Suite v5.12 software (Life Technologies). Reads were aligned with the hg19 human reference genome, and potential mutations were identified using Variant Caller v5.12. Raw variant calls were manually checked with the integrative genomics viewer (IGV; Broad Institute, Cambridge, MA, USA). Germline mutations were excluded using the Human Genetic Variation Database (https://www.hgvd.genome.med.kyoto‐u.ac.jp/).

### Immunohistochemistry (IHC)

FFPE was sectioned into 3‐μm slices and stained using the VENTANA BenchMark ULTRA automatic staining system (Roche, Basel, Switzerland) with β‐catenin (clone: β‐Catenin‐1, Dako, Glostrup, Denmark, setting: CC2 30 min, PAb 32 min). The staining was considered positive or negative based on the presence or absence of nuclear staining. Additionally, to assess the status of mismatch repair (MMR) proteins, staining was performed for MLH1 (clone: M1, Roche, setting: CC1 64 min, PAb 24 min), MSH2 (clone: G219‐1129, Roche, setting: CC1 40 min, PAb 24 min), MSH6 (clone: SP93, Roche, setting: CC1 64 min, PAb 12 min), PMS2 (clone: A16‐4, Roche, setting: CC1 92 min, PAb 32 min, Amp (4 min–4 min)). Samples with loss of expression in any of the proteins were classified as mismatch repair deficient (dMMR), while those with expression retained in all proteins were classified as mismatch repair proficient (pMMR). All IHC results were evaluated by T.O., a gynecologic pathologist.

### Droplet digital PCR

Using DNA extracted via LCM as described above, ddPCR was performed on eight hotspots of three genes [*CTNNB1* (D32A, D32N, S33C, or T41A), *PIK3CA* (E545K or H1047R), or *KRAS* (G12D or G12V)], and the variant allele frequency (VAF) was measured as previously reported [32]. Briefly, a 22‐μL reaction mixture was prepared containing 1.1 μL of PrimePCR for ddPCR *CTNNB1* (D32A, D32N, S33C, or T41A), *PIK3CA* (E545K or H1047R), or *KRAS* (G12D or G12V) (Bio‐Rad Laboratories, Hercules, CA, USA), 11 μL of ddPCR supermix for probes (no dUTP) (Bio‐Rad Laboratories), 5.9 μL of distilled water, and 4 μL of DNA. Droplets were prepared using an Automated Droplet Generator (Bio‐Rad Laboratories). After amplification in a thermal cycler, the copy numbers of wild‐type and mutant variants were counted using the QX200 Droplet Digital PCR System (Bio‐Rad Laboratories) at 20 μL per sample. VAFs were calculated as the ratio of mutant copies to total mutant and wild‐type copies. A VAF of 15% was used as a descriptive reference for enriched mutation signals, based on a prior study of cancer‐associated mutations in endometriosis and normal endometrium [6] and applied in our previous work [32]; it served as an operational reference, not a biological cutoff. Because VAF reflects the sampled mutant‐cell fraction and copy number state, signals below 15% were not regarded as negative, nor were signals ≥15% taken as evidence of compartment‐specific mutation.

Positive and negative controls were included in each run. Because the amount of DNA recovered from microdissected samples was below reliable quantification by standard spectrophotometry, input adequacy was assessed primarily from the total copy number obtained by ddPCR (with the total accepted droplets reported for reference), rather than from a measured DNA input; per‐reaction values are provided in Table S1. Owing to the limited material, replicate reactions were not feasible, and each sample was analyzed in a single well. Each sample was assayed for all eight hotspots. A reaction yielding a total copy number (mutant plus wild‐type) of 2.5 or fewer was considered to reflect negligible amplifiable template and was recorded as not applicable (NA). A sample was regarded as having failed quality control when three or more of the eight assays met this criterion, or when the positive or negative control did not perform as expected; samples with two or fewer such assays were retained for analysis.

### WES, somatic short variant calling, and allele‐specific copy number analysis

Reads from targeted amplicon sequencing were aligned to hg19, whereas whole‐exome sequencing reads were aligned to GRCh38. For two OEC cases and five SEOC cases, DNA extracted via macrodissection was used to perform library preparation with SureSelect XT HS2, Human All Exon V6 (Agilent Technologies, Santa Clara, CA, USA), followed by WES using NovaSeq 6000 (Illumina, San Diego, CA, USA). Because WES was limited to selected cases, it was not used for comprehensive cohort‐wide phylogenetic reconstruction. Raw FASTQ files were preprocessed using fastp (v0.23.2) [33] with default settings. The preprocessed reads were then aligned to the GRCh38 reference genome obtained from the GATK resource bundle using BWA (v0.7.17) [34]. The resulting BAM files underwent duplicate marking with Picard (v3.2.0) (https://broadinstitute.github.io/picard) and base quality score recalibration following GATK Best Practices using GATK (v4.4.0.0) [35]. SNVs and small insertions and deletions (indels) were identified using Mutect2 [36] with relevant data files from the GATK resource bundle. Variants labeled as ‘PASS’ in the FILTER column of VCF files were retained for downstream analysis. Allele‐specific copy number variations (CNVs), as well as tumor purity and ploidy estimations, were conducted using ASCAT v3.1.2 [37] in tumor‐normal paired mode with default settings using the reference files provided for processing next generation sequencing (NGS) data (ascatNGS). DNA samples were collected from three UEC cases and classified into four categories: tumor, tumor‐adjacent stroma, endometrial hyperplasia, and endometrial hyperplasia‐adjacent stroma. These samples were sequenced and analyzed using the same method as previously reported [38]. Briefly, libraries were prepared using SureSelect Human All Exon V4 or V5 (Agilent Technologies, Santa Clara, CA). WES was performed using HiSeq2500 or HiSeq2000 (Illumina, San Diego, CA). Sequence reads were aligned using BWA. GATK was used to recalibrate variant quality scores. Additionally, SNVs were detected using VarScan v2.3.7 [39], MuTect v1.1.4 [36], and Karkinos v3.0.22 [40]. Indels were identified using VarScan v2.3.7, SomaticIndelDetector v1.5–30 [35], and Karkinos v3.0.22. SNVs and indels were considered genuine mutations if they were detected by at least two of the three callers.

### Clonal relatedness

Clonal relatedness between anatomically distinct lesions was assessed based on shared cancer‐associated mutations detected by ddPCR, targeted sequencing, or WES. Because hotspot‐based assays do not capture genome‐wide phylogenetic relationships, shared hotspot mutations were interpreted as molecular evidence supporting clonal relatedness rather than definitive proof of monoclonality.

### Statistical analysis

Statistical analysis was performed using GraphPad Prism v10.4.1 (GraphPad Software, San Diego, CA, USA). Z‐tests were used to compare ratios between groups, Kruskal‐Wallis tests were used to compare VAFs, and Pearson's correlation coefficient was used to assess the correlation between macrodissection‐based sequencing VAFs and mean ddPCR‐based VAFs across cases. *P* < 0.05 was considered statistically significant.

## Results

### Clinical background of OEC and SEOC

The mean age of the 39 OEC cases was 54.8 ± 14.0 years. According to the FIGO 2008 staging system, 26 cases (66.7%) were Stage I, five cases (12.8%) were Stage II, and eight cases (20.5%) were Stage III. Histological grading revealed 22 cases (56.4%) as Grade 1, 10 cases (25.6%) as Grade 2, five cases (12.8%) as Grade 3, and two cases (5.1%) with mixed histological types. The MMR status was dMMR in five cases (12.8%). The mean follow‐up period was 74.1 ± 40.0 months, during which seven cases (17.9%) of recurrence and five cases (12.8%) of death were observed.

Among the 39 cases of OEC, eight cases of SEOC were included, with a mean age of 45.6 ± 12.5 years. Additionally, the histological grade was Grade 1 in seven cases (87.5%) and Grade 2 in one case (12.5%), with dMMR present in two cases (25%). No recurrence or death was observed in any of the eight cases during the observation period.

### Macrodissection and targeted sequencing of OEC

Figure 1 summarizes the somatic mutations identified by targeted amplicon sequencing in the 33 mutation‐positive cases among the 39 OEC cases. The most frequent mutations were *CTNNB1*: 13 cases (33.3%), *PIK3CA*: 13 cases (33.3%), *PTEN*: 13 cases (33.3%), *KRAS*: nine cases (23.1%), and *TP53*: eight cases (20.5%) (Fig. 1). The mutational spectrum was broadly consistent with previous reports [2, 14, 25]. Among the 13 cases with *CTNNB1* mutations, 10 cases (76.9%) were Stage I, and 12 cases (92.3%) had Grade 1 or 2 histology, excluding one case with coexisting *TP53* mutations (Fig. 1). Additionally, only one case (7.7%) recurred, and there were no deaths (Fig. 1). In contrast, *TP53*‐mutated cases were more advanced, with 62.5% being Stage III and 37.5% classified as Grade 3 or mixed histology, indicating high‐grade disease (Fig. 1). Recurrence and death were observed in three cases (37.5%) (Fig. 1).

**Fig. 1:**
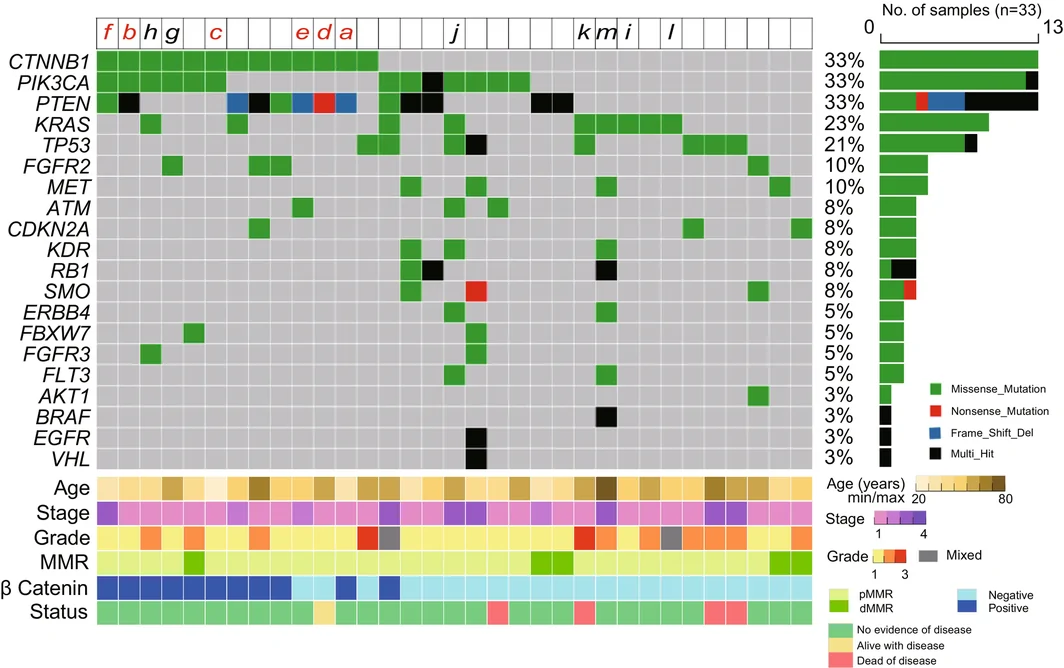
Clinicopathologic and mutational landscape of mutation‐positive ovarian endometrioid carcinomas among the 39‐case cohort. Cancer‐associated mutations identified by targeted amplicon sequencing of a single macrodissected (bulk) tumor sample per case are shown for the 33 mutation‐positive cases; spatially resolved, multiregion data are presented in Fig. 2. The 13 cases selected for ddPCR‐based spatial analysis are indicated by letters. Cases shown in red represent SEOC. The upper panel lists gene mutations in each case in order of frequency, with the frequencies also indicated. The lower panel uses a heatmap to show the clinical information of each case corresponding to the upper panel, including age, stage, tumor histology, mismatch repair status, β‐catenin immunohistochemistry findings, and prognosis. MMR: mismatch repair, dMMR: mismatch repair deficient, pMMR: mismatch repair proficient.

### Intratumor heterogeneity of *CTNNB1* , *PIK3CA* , and *KRAS* mutations

Next, we focused on *CTNNB1*, *PIK3CA*, and *KRAS*, which were frequently mutated and had mutation hotspots detected by targeted amplicon sequencing. Of the 39 OEC cases, 15 carried at least one assayable hotspot mutation in *CTNNB1* (D32A, D32N, S33C, or T41A), *PIK3CA* (E545K or H1047R), or *KRAS* (G12D or G12V) (Fig. 1); the remaining 24 cases were excluded because no targeted hotspot mutation was present. Of these 15 eligible cases, two were further excluded because of insufficient or poor‐quality FFPE tissue for LCM, leaving 13 cases (including six SEOC cases) for LCM‐ddPCR analysis. For these cases, we performed LCM on tumor epithelial regions (each containing approximately 20–30 cells) and on tumor‐adjacent and tumor‐distant stromal regions. A total of 166 tumor‐epithelial, 66 tumor‐adjacent stromal, and 22 tumor‐distant stromal samples were collected. Of these, 139, 32, and 20 samples, respectively, met the sample‐level QC criteria and were included in the analysis. The 139 tumor epithelial samples comprised 92 from ovarian and 47 from endometrial tumors; the 32 tumor‐adjacent stromal samples comprised 19 ovarian and 13 endometrial; and the 20 tumor‐distant stromal samples comprised 16 ovarian and 4 endometrial. VAFs were measured by ddPCR (Fig. 2). There was a strong positive correlation between the VAF detected by targeted amplicon sequencing of macrodissection samples and the average VAF detected by LCM with ddPCR in tumor epithelial regions (*r* = 0.86, *P* = 0.0026) (Fig. S3).

**Fig. 2:**
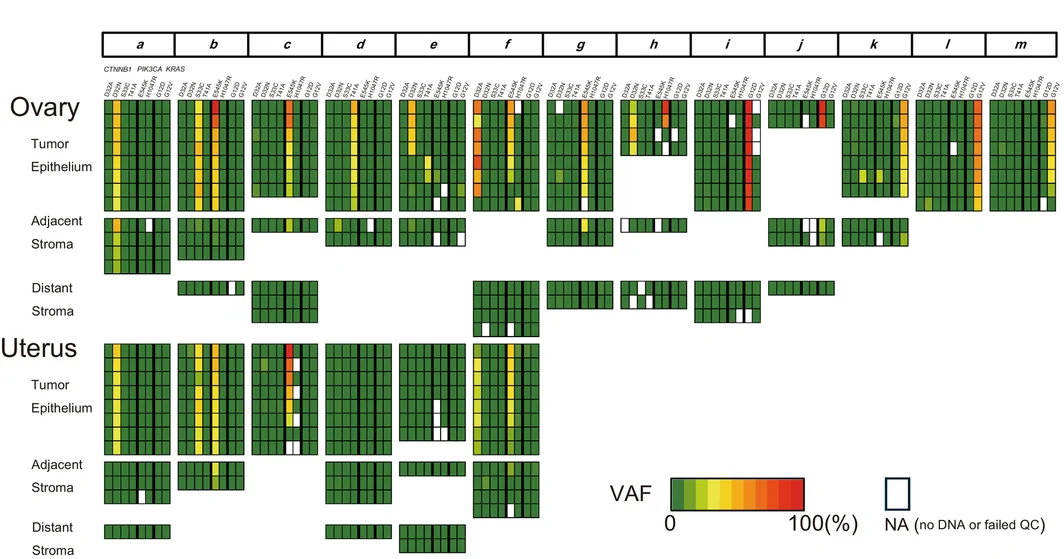
Spatial distribution of hotspot mutations assessed by droplet digital PCR in microdissected samples from 13 cases. Each row represents one microdissected sample. Samples are grouped as tumor epithelium, tumor‐adjacent stroma, and tumor‐distant stroma. Columns indicate hotspot mutations in *CTNNB1*, *PIK3CA*, and *KRAS* (*CTNNB1* D32A, D32N, S33C, and T41A, followed by *PIK3CA* E545K, H1047R, and *KRAS* G12D and G12V). In SEOC cases, ovarian and endometrial lesions are shown separately. This figure illustrates spatial heterogeneity of hotspot mutation signals across microdissected tumor epithelial regions and stromal compartments. In several cases, mutation‐positive and mutation‐negative epithelial regions coexisted within the same tumor, whereas tumor‐distant stromal compartments were negative for the assayed hotspot mutation signals. Blank cells indicate individual assays with negligible amplifiable template or samples that failed sample‐level quality control. VAF: variant allele frequency, NA: not applicable.

Uniformly high VAFs across sampled regions were observed only in a subset of cases, whereas most tumors contained both mutation‐positive and mutation‐negative epithelial regions, indicating marked spatial intratumor heterogeneity (Fig. 2).

Additionally, among the six SEOC cases, common hotspot mutations were identified in both ovarian tumor epithelium and endometrial tumor epithelium in four cases (cases *a*, *b*, *c*, and *f*), but no common hotspot mutations were detected in the remaining two cases (cases *d* and *e*) (Fig. 2).

### Relationship between *CTNNB1* mutations and β‐catenin nuclear positivity by IHC

β‐catenin nuclear positivity was observed in 11 of 39 cases (28.2%), and *CTNNB1* mutations were identified in 13 of 39 cases (33.3%) by targeted amplicon sequencing. Both were present in 10 cases (Fig. 1). As previously reported [41, 42], the regions where nuclear β‐catenin was positive were primarily morules. In the three cases where *CTNNB1* mutations were identified by sequencing but were IHC‐negative, morules were not detected (Fig. S4A).

In case *f*, LCM with ddPCR revealed intratumor heterogeneity of the *CTNNB1* D32A mutation. In morules positive for β‐catenin IHC, the *CTNNB1* D32A mutation was detected by ddPCR (Fig. S4B). On the contrary, in IHC‐negative areas, there were regions where the *CTNNB1* D32A mutation was detected (Fig. S4C) and regions where it was not detected (Fig. S4D).

### Clonal relatedness pattern in SEOC

Whole‐exome sequencing was performed on five of the six SEOC cases (cases *a*, *b*, *d*, *e*, and *f*). In four of these (cases *a*, *b*, *d*, and *f*), multiple common genetic mutations were detected in both endometrial and ovarian tumors, consistent with previous reports [17, 18, 19], and these findings supported the existence of a clonal relationship between endometrial and ovarian lesions (Table S2).

Using the LCM‐ddPCR profiles, we examined the distribution of shared and distinct hotspot mutations between the endometrial and ovarian lesions in these four cases (Fig. 3). SEOCs are generally considered to represent dissemination from the endometrium to the ovary [7, 9, 10], and our observations are consistent with this model; however, given the limited WES sampling and possible unsampled subclones, the present mutation data do not by themselves establish directionality in individual cases. In cases *a* and *b*, a dominant hotspot mutation was shared at comparable VAFs in both lesions, consistent with a common clonal origin. In case *d*, an oncogenic hotspot mutation was present in the ovarian lesion but absent from the sampled endometrial regions, which may reflect a spatially restricted or unsampled subclone. In case *f*, all endometrial regions shared *CTNNB1* D32A and *PIK3CA* E545K, whereas the ovarian regions were heterogeneous: most carried both mutations, while one region harbored *CTNNB1* D32A alone and another harbored *PIK3CA* H1047R alone, indicating multiple subclonal populations within the ovarian tumor.

**Fig. 3:**
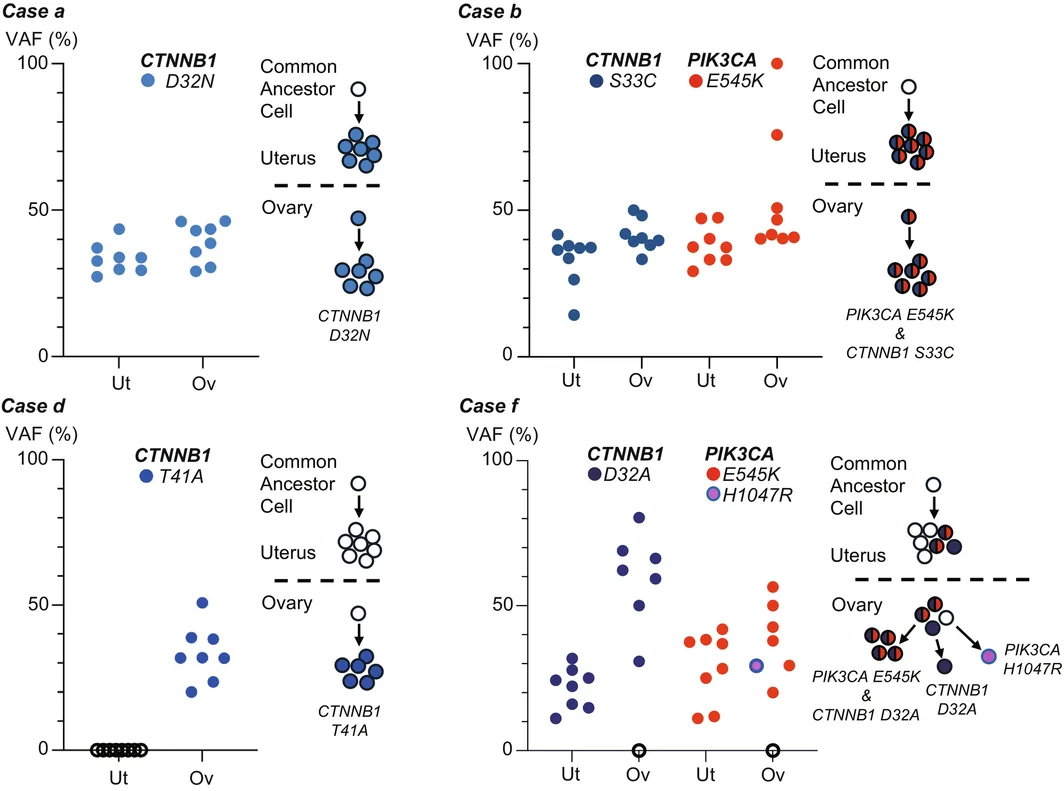
Gene mutations, variant allele frequencies, and inferred clonal relatedness models in tumors of synchronous endometrial and ovarian carcinomas (SEOC). This figure shows the four SEOC cases (cases *a*, *b*, *d*, and *f*) in which shared somatic mutations were identified in both uterine and ovarian tumors by whole‐exome sequencing (four of the five SEOC cases analyzed by WES). In the scatter plots, each point represents one microdissected sample; VAFs are shown for the endometrial and ovarian tumors. The schematics illustrate the inferred clonal relatedness between the uterine and ovarian lesions based on the shared and distinct hotspot mutations. Ut: uterus, Ov: ovary, VAF: variant allele frequency.

### Detection of hotspot mutation signals in microdissected adjacent stromal compartments

In selected cases, ddPCR detected low‐level hotspot mutation signals in microdissected stromal compartments adjacent to tumor glands. These signals were not uniformly detected across all stromal samples and were generally lower than those observed in corresponding tumor epithelial compartments. In contrast, no targeted hotspot mutation signals were detected in the distant stromal compartments (Fig. 2). Hotspot mutation signals with VAF ≥ 15% [6] were detected in the adjacent stroma in 6 of 10 cases of ovarian or endometrial tumor stroma evaluated by LCM using ddPCR; in five of these cases, excluding case *d*, the same hotspot mutation signals were detected in both the tumor epithelial and adjacent stromal compartments (Fig. 4; postmicrodissection images of these adjacent stromal regions are shown in Fig. S5). In case d, the adjacent stromal sample with a detectable mutation signal was obtained from an area adjacent to epithelium that carried the *CTNNB1* T41A mutation but not the D32N mutation. However, the mutation detected in the adjacent stroma was the *CTNNB1* D32N mutation, suggesting that it may not have originated from nearby tumor cells (Figs 2, 4).

**Fig. 4:**
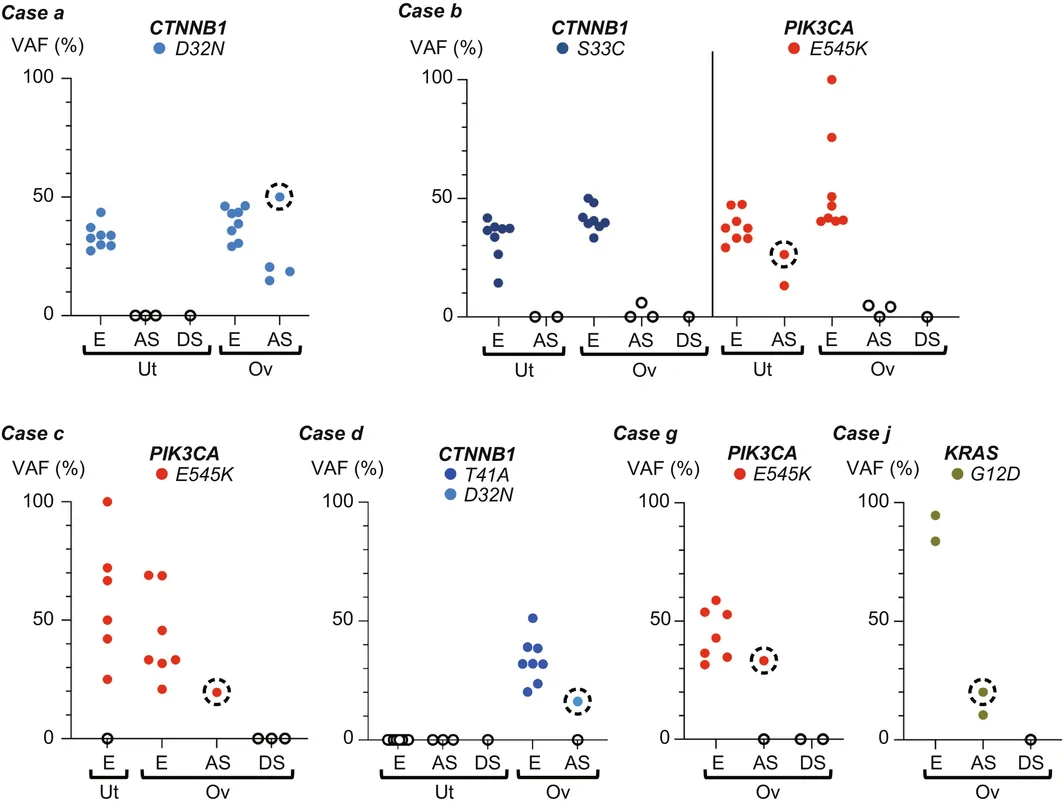
Variant allele frequency (VAF) in tumors, tumor‐adjacent stroma, and distant stroma. In cases of synchronous endometrial and ovarian tumors, hotspot mutations are shown separately for the uterus and ovary. In the six cases shown, hotspot mutation signals with VAFs ≥ 15% were detected in tumor‐adjacent stromal compartments. Furthermore, in five of six cases, the same hotspot mutation signal was detected in both the tumor epithelial and adjacent stromal compartments. However, in one case, the mutation detected in the adjacent stroma differed from that in the tumor epithelium (*CTNNB1* D32N). Adjacent stromal samples with hotspot mutation signals at VAFs ≥ 15% are indicated by dashed circles; the corresponding postmicrodissection [laser capture microdissection (LCM)] images of these adjacent stromal regions are shown in Fig. S5, confirming that the sampled stromal compartments were morphologically distinct from, and excluded, overt tumor epithelium. This 15% VAF value was used as a descriptive reference and not as a biological cutoff. E: tumor epithelium, AS: adjacent stroma, DS: distant stroma, Ut: uterus, Ov: ovary.

### Genetic mutations in endometrial cancer, endometrial hyperplasia, and the adjacent stromal regions

In three cases of endometrial endometrioid carcinoma with concomitant endometrial atypical hyperplasia, four regions—the cancer epithelial area and its adjacent stromal area, and the hyperplasia area, and its adjacent stromal area—were sampled via macrodissection and subjected to WES. The results showed that somatic mutations identified in the cancer epithelial area were also detected at lower VAFs in the adjacent stromal area in all three cases (Fig. 5). However, the VAF of common genetic mutations was significantly lower in the adjacent stromal area in all three cases (*P* < 0.0001 in all three cases) (Fig. 5). Additionally, the proportions of gene mutations shared between the cancer epithelial area and its adjacent stromal area were significantly higher than those shared between the hyperplasia area and its adjacent stromal area (*P* = 0.0005, *P* = 0.0022, *P* < 0.0001, respectively) (Fig. 5).

**Fig. 5:**
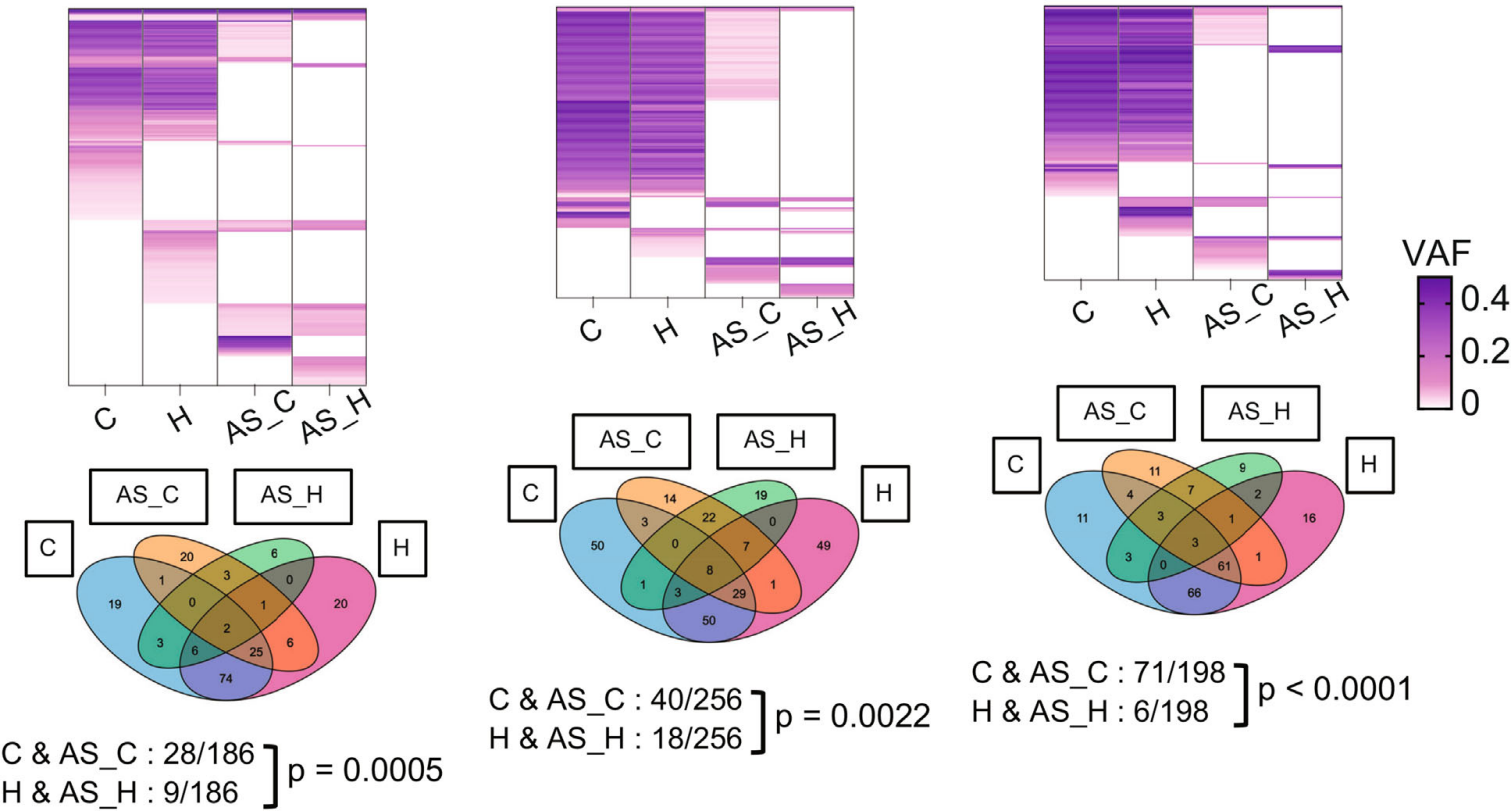
To further explore whether mutation sharing between epithelial lesions and adjacent stromal compartments may also be observed in endometrioid tumors of the uterus, we analyzed three uterine endometrioid carcinoma (UEC) cases with concomitant atypical hyperplasia. Whole‐exome sequencing reveals shared somatic mutations between endometrial carcinoma and adjacent stroma, but fewer shared mutations between endometrial hyperplasia and adjacent stroma. C: cancer, H: endometrial hyperplasia, AS_C: adjacent stroma from cancer, AS_H: adjacent stroma from endometrial hyperplasia, VAF: variant allele frequency.

### *KRAS* mutations with high VAF

The two cases (cases *i* and *j*) with the *KRAS* G12D mutation had tumor epithelial VAFs ranging from 83.7% to 100% by LCM, uniformly exceeding 50% (Fig. 2). Additionally, WES‐based CNV analysis in these two cases revealed loss of heterozygosity (LOH) of *KRAS* in case *i* and fivefold amplification of one allele in the *KRAS* region in case *j*. Notably, such *KRAS* region LOH or amplification was not observed in three cases of endometrial hyperplasia with endometrioid carcinoma or five cases of SEOC.

## Discussion

Oncogenic hotspot mutations are often assumed to be clonally ubiquitous within tumors. The *CTNNB1*, *PIK3CA*, and *KRAS* hotspots assessed here are well‐established oncogenic drivers [43, 44, 45, 46, 47, 48, 49, 50, 51]; nevertheless, our findings indicate that, for these selected hotspots, the corresponding mutation signals were not uniformly distributed across all sampled tumor regions and could exhibit marked spatial restriction. In cases where mutations were detected by macrodissection, LCM analysis nonetheless revealed tumor epithelial regions without detectable signals for the corresponding hotspot mutations (Fig. 2). This discrepancy mirrors our previous observations in OCCC [32]. Furthermore, our findings suggest that at least a subset of SEOC classified by clinicopathological criteria may share molecular alterations consistent with clonal relatedness (Table S2). However, the assayed hotspot profiles differed between the uterine and ovarian lesions in some SEOC cases, indicating inter‐organ heterogeneity in these selected hotspots (Figs 2, 3). The presence of sampled tumor regions with and without detectable signals for the assessed hotspots suggests that single‐region biomarker assessment may incompletely represent regional hotspot profiles. Accordingly, assessment of intratumor genetic heterogeneity may help refine biomarker interpretation in endometrioid carcinomas. In addition, β‐catenin immunohistochemistry showed nuclear positivity predominantly in morules, and *CTNNB1*‐mutant regions lacking morular differentiation were often IHC‐negative. This indicates that mutation‐positive, IHC‐negative regions most likely reflect nonmorular *CTNNB1*‐mutant cells in which nuclear β‐catenin does not accumulate, although limited IHC sensitivity and spatial mismatch between the ddPCR and immunohistochemistry sections may also contribute.

Previously, we identified *PIK3CA* mutations identical to those in tumor epithelium in the tumor stroma of OCCC [32]. Consistently, we detected hotspot mutation signals shared with tumor epithelium in adjacent stromal compartments of OEC (Figs 2, 4). Similarly, Narikiyo et al. reported cases of OEC where the same *KRAS* mutation was identified in both tumor epithelium and stroma [52]. Mullerian endometrioid carcinoma is sometimes accompanied by the development of coexisting sarcomas, resulting in carcinosarcoma [53, 54]. In such tumors, the relationship between their tumor epithelium and stroma may be closer than that in other tumor types. A previous study showed that side‐population cells of endometrial cancer can differentiate into mesenchymal cells [55]. In contrast, few common genetic mutations were identified in endometrial atypical hyperplasia, a precancerous lesion without invasive potential, and its adjacent stroma (Fig. 5). The detection of hotspot mutation signals in these adjacent stromal compartments should be interpreted cautiously. The most likely explanation is minimal inclusion of epithelial (tumor) cells during microdissection; other possibilities include tumor cells undergoing partial epithelial–mesenchymal transition, tumor‐derived DNA in the stromal microenvironment, or technical artifacts related to low‐input DNA analysis. Because the present data cannot establish the cellular origin of these low‐level signals, we refrain from attributing biological significance to them and regard them as exploratory observations requiring orthogonal validation.

In the current OEC analysis, *KRAS*‐mutant tumors showed uniformly high VAFs, and WES suggested LOH or amplification at the *KRAS* locus. These findings indicate that, in selected OECs, *KRAS*‐mutant clones may become dominant in association with allele‐specific copy number imbalance.

Spatially resolved molecular data for OEC remain limited compared with other endometriosis‐associated and high‐grade ovarian histotypes. Multiregion whole‐genome sequencing has demonstrated extensive spatial subclonal diversity in high‐grade serous ovarian carcinoma [56] and punctuated evolution with substantial intratumor heterogeneity in OCCC [57]. Because these studies reconstructed genome‐wide clonal architecture, whereas our analysis was restricted to selected hotspot mutations interrogated by LCM‐ddPCR and targeted amplicon sequencing, the absolute degree of intratumor heterogeneity cannot be directly compared across histotypes. Qualitatively, however, the spatial heterogeneity of selected hotspot mutations observed here in OEC is consistent with the subclonal diversity reported in these related histotypes, and the relative scarcity of spatially resolved data for OEC was one motivation for the present study.

This study has several limitations. First, endometrioid ovarian cancer is a relatively rare disease, and because this study was conducted retrospectively at a single institution, the cohort size was limited. In addition, the LCM‐ddPCR cohort was predominantly composed of Grade 1 tumors (9 of 13 cases; Fig. 1), which precluded a statistically meaningful comparison of spatial heterogeneity across histological grades; a grade‐dependent difference in intratumor heterogeneity remains plausible and warrants investigation in a larger, grade‐balanced cohort. Furthermore, *POLE* mutation analysis was attempted but failed quality control in the available archival FFPE, and *MLH1* promoter methylation was not assessed in MMR‐deficient cases; consequently, neither the *POLE*‐ultramutated subtype nor the sporadic‐versus‐hereditary distinction could be resolved, limiting integration with current molecular classification. This limitation is most relevant to the SEOC cases, in which subtype concordance between the endometrial and ovarian lesions would further inform clonal relatedness. Second, whole‐exome sequencing was performed only on selected cases and was used as an exploratory method to complement hotspot‐based spatial profiling, rather than as a phylogenetic approach targeting the entire cohort. Third, detection of hotspot mutations via ddPCR does not allow for complete reconstruction of clonal relationships across the entire genome; therefore, shared hotspot mutations should be interpreted as evidence supporting clonal relationships rather than definitive proof of monoclonality. In addition, ddPCR‐derived VAFs do not account for local copy number or ploidy differences, which can affect VAF independently of the proportion of mutant cells; allele‐specific copy number was assessed only in cases analyzed by WES. The spatial analysis also targeted only these selected hotspots, and other frequently altered genes such as *PTEN*, *ARID1A*, *TP53*, and *POLE* were not interrogated spatially. Fourth, mutation signals in microdissected stromal samples were generally low‐level and require cautious interpretation, particularly because of the low DNA input. Orthogonal validation was not feasible here: replicate LCM‐ddPCR was precluded by the limited remaining material, and currently available spatial methods cannot reliably resolve these specific hotspots on archival FFPE [20, 21]. These signals should therefore be regarded as exploratory. Finally, this study did not perform functional validation of the biological effects of spatially heterogeneous mutations.

## Conclusion

In conclusion, our study reveals marked spatial heterogeneity of selected *CTNNB1*, *PIK3CA*, and *KRAS* hotspot mutation signals in OEC and supports clonal relatedness in a subset of SEOC. These findings highlight that single‐region genotyping may underestimate spatially restricted hotspot profiles and underscore the value of multiregion molecular assessment in ovarian endometrioid carcinoma.

## Conflict of interest

Kosuke Murakami received a research grant from Eisai. Noriomi Matsumura received honoraria from Chugai Pharmaceutical, AstraZeneca, and Takeda Pharmaceutical and a research grant from AstraZeneca, and serves as an outside director of Takara Bio. The authors declare no other competing financial interests relevant to this work.

## Author contributions

TA, KM, and NM conceptualized the study. KM, ST, SM, and KS developed the methodology. TA, KM, and AK conducted the investigation. TA, KM, ST, SM, and KS performed the formal analysis. TA and ST curated the data. AK provided resources. TO and YK performed validation. TO conducted the pathological evaluation. TA prepared the visualizations and wrote the original draft. KM, KN, KA, and NM supervised the study. KM administered the project. NM acquired funding. KM, ST, AK, SM, TO, KS, YK, KN, KA, and NM reviewed and edited the manuscript.

## Supporting information


**Fig. S1.** Research scheme and case‐selection flow.
**Fig. S2.** Laser capture microdissection of tumor epithelial and adjacent stromal compartments in case d.
**Fig. S3.** Correlation of variant allele frequencies (VAFs) obtained by targeted amplicon sequencing of macrodissected tumor tissue and by droplet digital PCR (ddPCR) of laser capture microdissected samples.
**Fig. S4.** Relationship between CTNNB1 mutations identified by targeted amplicon sequencing and β‐catenin immunohistochemical staining, with specific examples.
**Fig. S5.** Postmicrodissection (laser capture microdissection) images of the mutation‐positive adjacent stromal regions.

**Table S1.** Droplet digital PCR data for laser capture microdissected tumor epithelial, tumor‐adjacent stromal, and tumor‐distant stromal compartments from ovarian and endometrial tumors.

**Table S2.** Common genetic mutations in both endometrial and ovarian tumors.

## Data Availability

The sequencing data generated in this study are not publicly available due to restrictions related to patient privacy and institutional regulations. However, de‐identified data are available from the corresponding author upon reasonable request.
