# Supplementary material for: Spatial heterogeneity of hotspot mutations and clonal relatedness in ovarian endometrioid carcinoma
Source: Mol Oncol. 2026 Oct 8:10.1002/1878-0261.70347. Online ahead of print. doi: 10.1002/1878-0261.70347 (PMC13648112; doi:10.1002/1878-0261.70347)
Supplement: Supplementary file 1 [file MOL2-9999-0-s002.pdf]

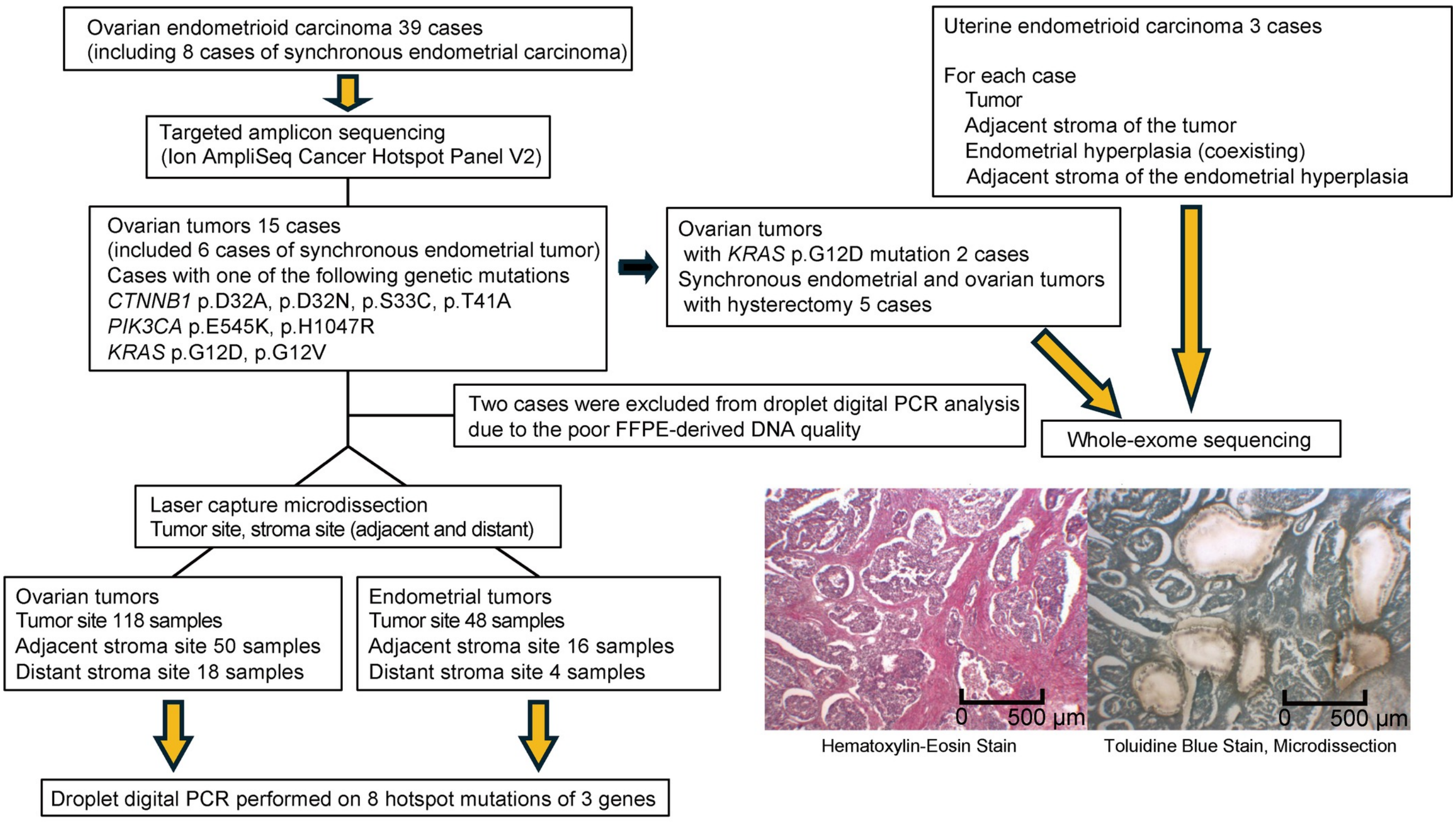

Supplementary Figure S1. Research scheme and case-selection flow. Targeted amplicon sequencing was performed on 39 cases of ovarian endometrioid carcinoma (including 8 cases with concurrent endometrial cancer). Of these, 15 cases carried at least one assayable hotspot mutation in CTNNB1, PIK3CA, or KRAS; 24 cases were excluded because no targeted hotspot mutation was present, and a further 2 cases were excluded because of insufficient or poor-quality FFPE tissue, leaving 13 cases for laser capture microdissection and droplet digital PCR. Whole-exome sequencing was additionally performed for 3 separate cases of endometrial cancer. Representative hematoxylin-eosin- and toluidine blue-stained micrographs illustrating laser capture microdissection are shown in the lower right. Scale bars: 500 μm.

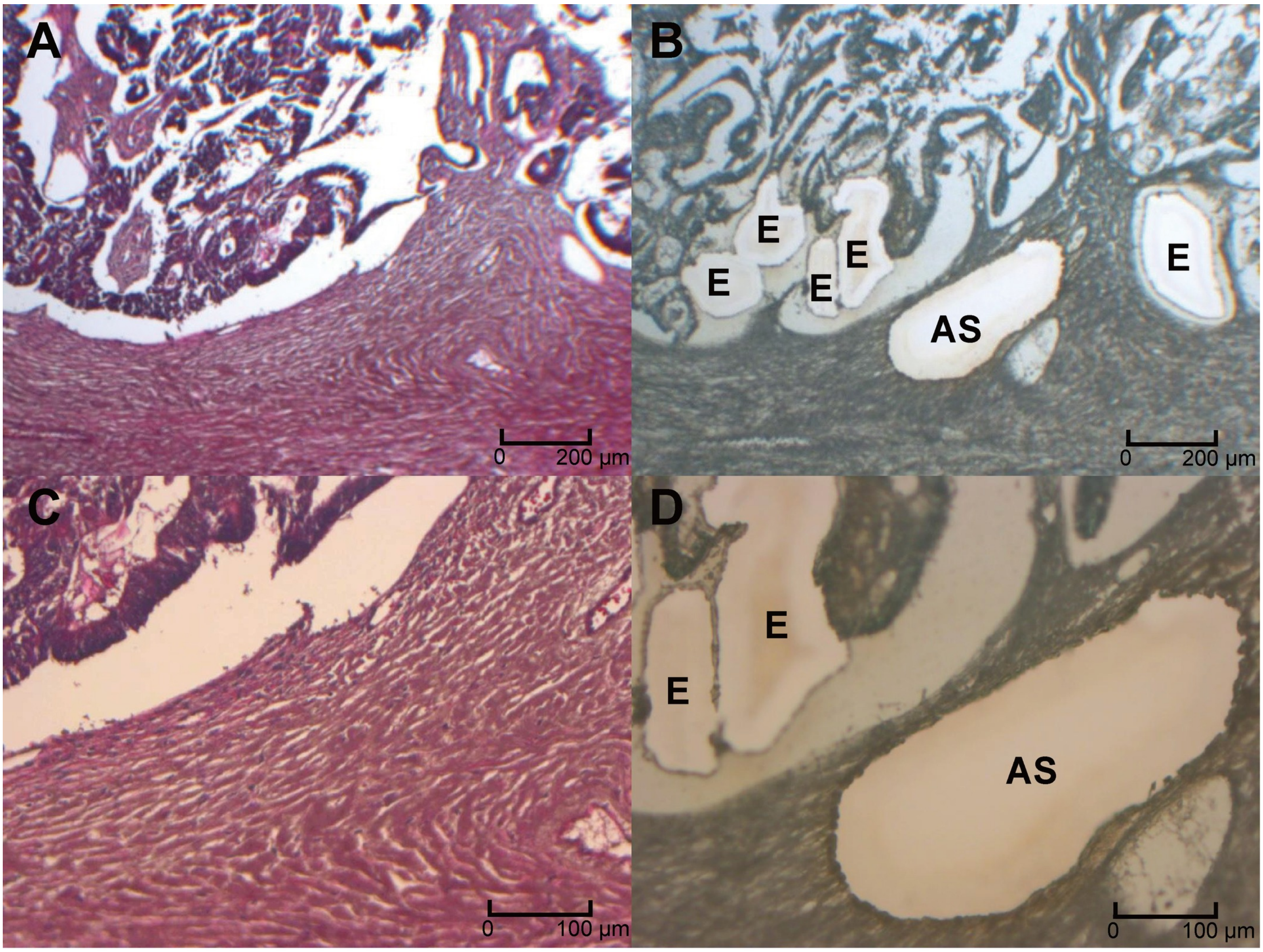

Supplementary Figure S2. Laser capture microdissection of tumor epithelial and adjacent stromal compartments in case d. (A) Hematoxylin and eosin-stained serial section. (B) Corresponding toluidine blue-stained section after LCM. (C, D) Higher-magnification views of the regions shown in (A) and (B), acquired separately with higher-power objectives rather than by digital enlargement; the differences in brightness and background appearance therefore reflect acquisition conditions rather than post-acquisition processing. The case d panel of Supplementary Figure S5 is a cropped overview of the same source image as panel B, and panel D shows the same post-LCM sampling site at higher magnification; this site is intentionally shown in both figures and is not presented as an independent region or replicate. Scale bars: 200 µm (A, B); 100 µm (C, D). AS: adjacent stroma, E: epithelium, LCM: laser capture microdissection.

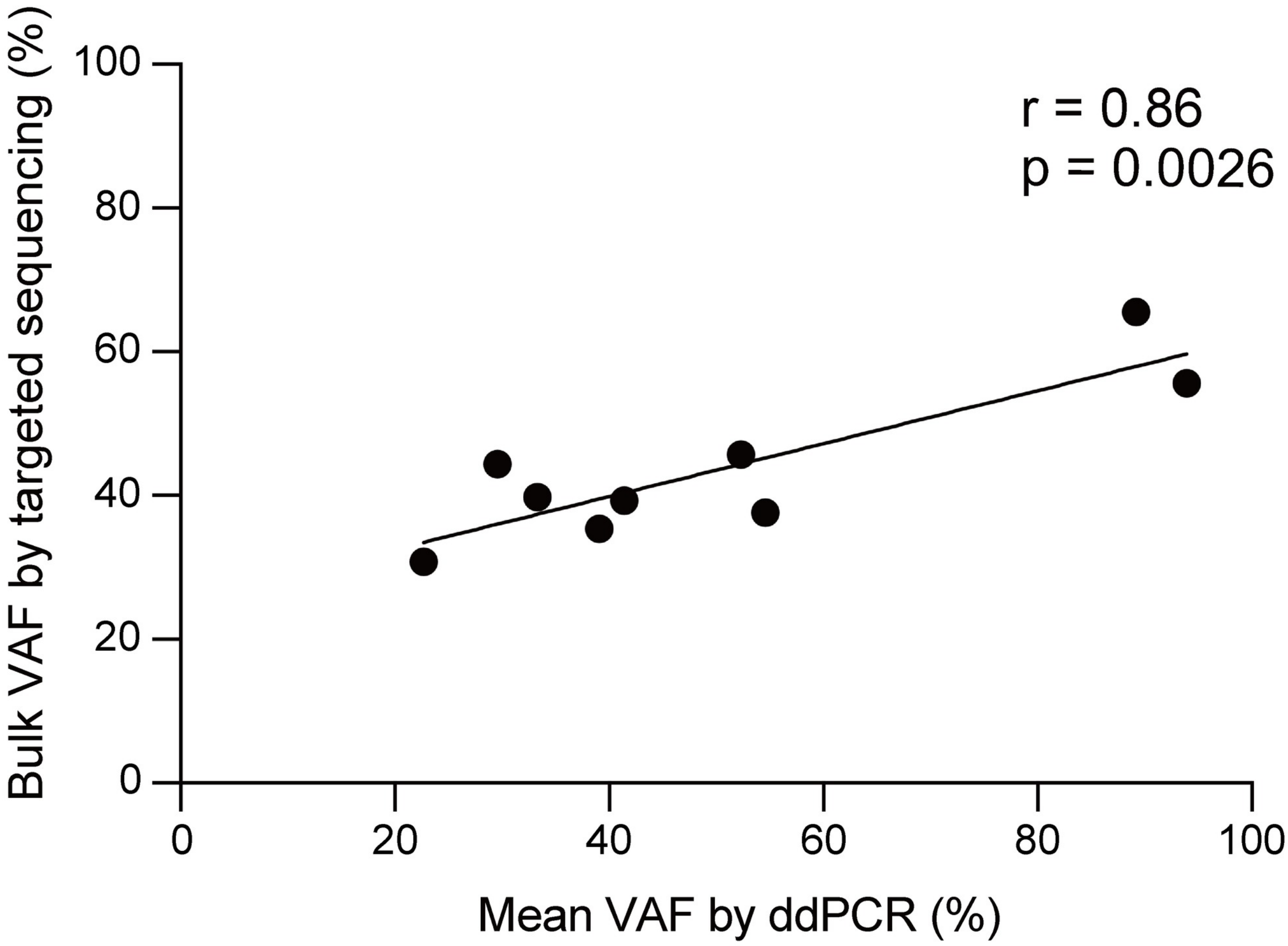

Supplementary Figure S3. Correlation of variant allele frequencies (VAFs) obtained by targeted amplicon sequencing of macrodissected tumor tissue and by droplet digital PCR (ddPCR) of laser capture microdissected samples. This figure shows the correlation results comparing VAF of CTNNB1, PIK3CA, and KRAS mutations obtained by different methods in 9 cases of ovarian endometrioid carcinoma. The association between targeted-sequencing VAF and mean ddPCR VAF was evaluated using a two-sided Pearson correlation test ($r = 0.86$, $P = 0.0026$). VAF: variant allele frequency, ddPCR: droplet digital PCR.

A

	CTNNB1 mutation (+)	*CTNNB1* mutation (-)
β-catenin IHC (+)	n=10	n=1
β-catenin IHC (-)	n=3 (no morules)	n=25

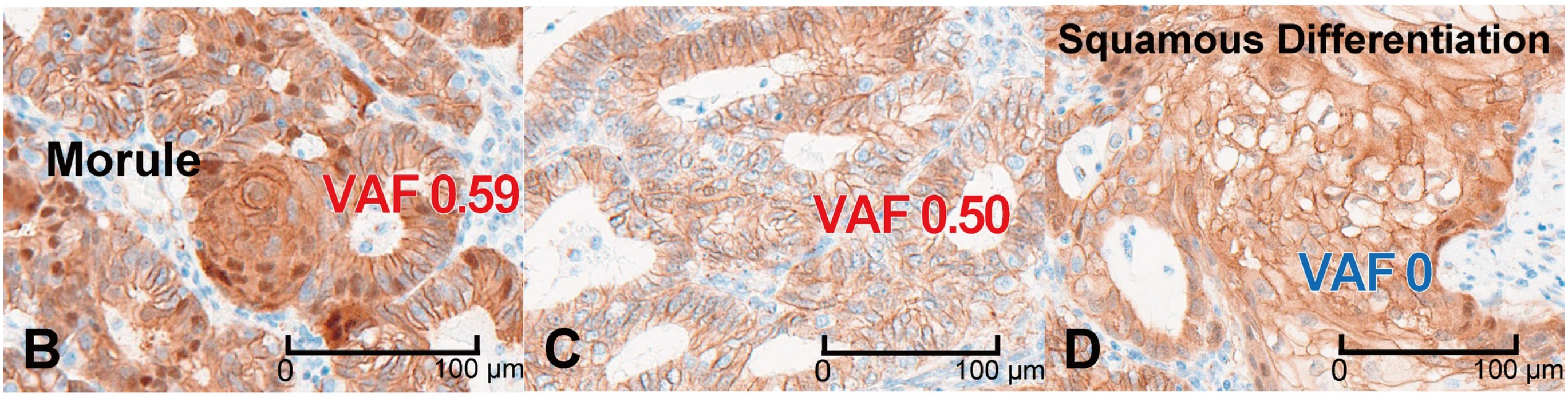

Supplementary Figure S4. Relationship between CTNNB1 mutations identified by targeted amplicon sequencing and β-catenin immunohistochemical staining, with specific examples. A. Classification based on the presence or absence of CTNNB1 mutations identified by targeted amplicon sequencing and β-catenin immunohistochemical staining in the 39 cases in this study, along with the number of cases. B. Representative image of a case positive for CTNNB1 mutations and β-catenin immunohistochemical staining. Nuclear staining is observed in morules. C. Representative image of a case positive for CTNNB1 mutations but negative for β-catenin immunohistochemical staining. No morules are observed in the slide. D. High-magnification image showing representative β-catenin immunohistochemical findings in squamous differentiation without CTNNB1 mutation signals. IHC: immunohistochemistry, VAF: variant allele frequency. Scale bars: 100 µm (B, C, D).

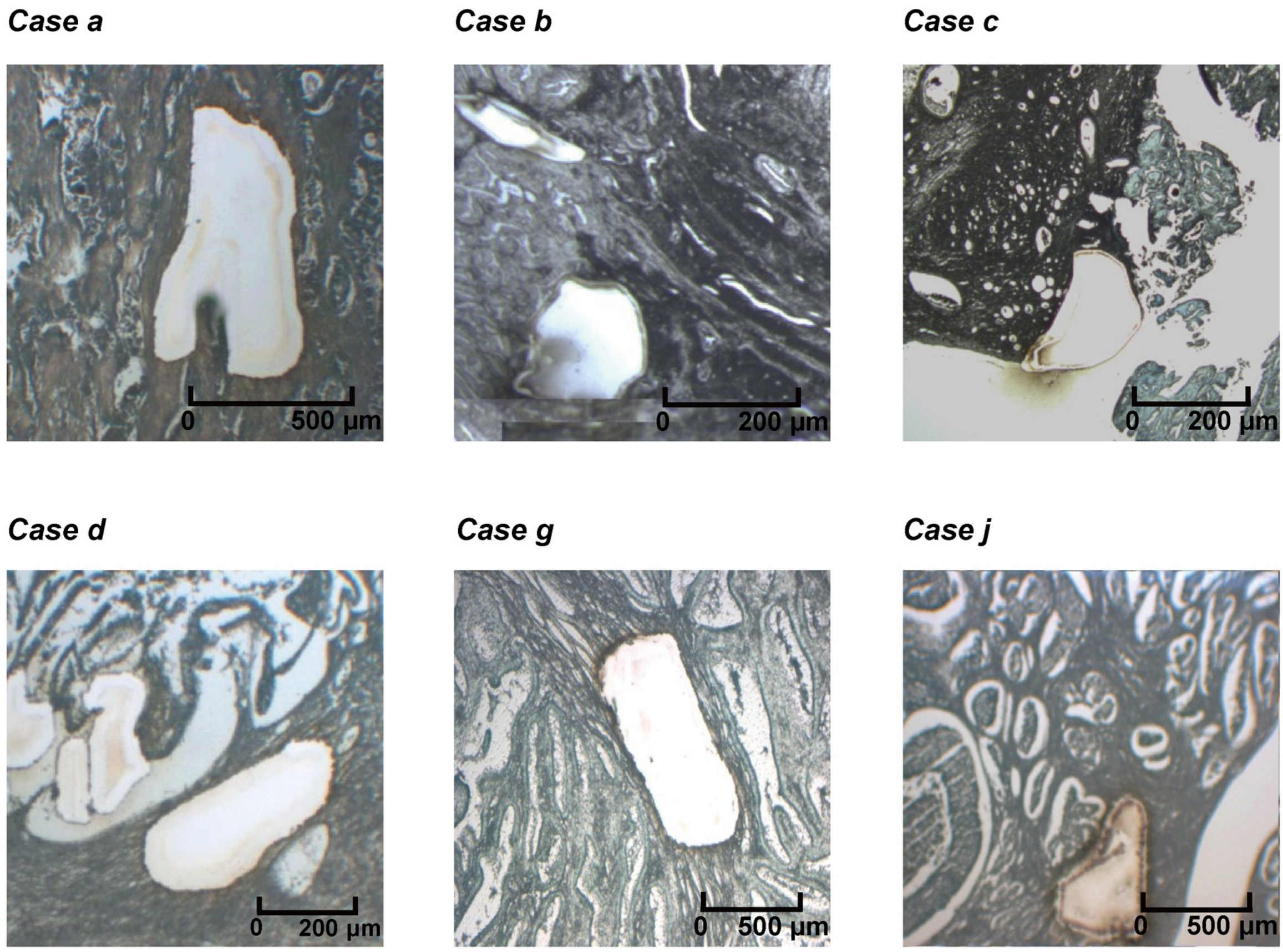

Supplementary Figure S5. Post-microdissection (laser capture microdissection) images of the mutation-positive adjacent stromal regions. For each case in which a hotspot mutation signal was detected in the tumor-adjacent stroma (cases a, b, c, d, g, and j; indicated by dashed circles in Figure 4), the toluidine blue–stained section after microdissection of the corresponding adjacent stromal region is shown. In each case, the microdissected stromal compartment was morphologically distinct from, and excluded, overt tumor epithelium. The case d panel is derived from the same source image as Supplementary Figure S2B. Supplementary Figure S2D shows the same post-LCM sampling site in a separately acquired higher-magnification image. These images do not represent independent sampling regions or replicates. Scale bars: 500 µm (cases a, g, j); 200 µm (cases b, c, d). LCM: laser capture microdissection.
